# Impact of Early Blood Pressure Lowering in Patients Presenting with Acute Ischemic Stroke

**DOI:** 10.1007/s11886-021-01497-0

**Published:** 2021-05-07

**Authors:** A. Maud, G. J. Rodriguez, A. Vellipuram, F. Sheriff, M. Ghatali, V. Gupta, R. Khatri, S. Cruz-Flores

**Affiliations:** Department of Neurology, Texas Tech University Health Sciences Center El Paso, El Paso, TX USA

**Keywords:** Blood pressure, Acute ischemic stroke, Cerebrovascular disease

## Abstract

**Purpose of Review:**

In this review article we will discuss the acute hypertensive response in the context of acute ischemic stroke and present the latest evidence-based concepts of the significance and management of the hemodynamic response in acute ischemic stroke.

**Recent Findings:**

Acute hypertensive response is considered a common hemodynamic physiologic response in the early setting of an acute ischemic stroke. The significance of the acute hypertensive response is not entirely well understood. However, in certain types of acute ischemic strokes, the systemic elevation of the blood pressure helps to maintain the collateral blood flow in the penumbral ischemic tissue. The magnitude of the elevation of the systemic blood pressure that contributes to the maintenance of the collateral flow is not well established. The overcorrection of this physiologic hemodynamic response before an effective vessel recanalization takes place can carry a negative impact in the final clinical outcome. The significance of the persistence of the acute hypertensive response after an effective vessel recanalization is poorly understood, and it may negatively affect the final outcome due to reperfusion injury.

**Summary:**

Acute hypertensive response is considered a common hemodynamic reaction of the cardiovascular system in the context of an acute ischemic stroke. The reaction is particularly common in acute brain embolic occlusion of large intracranial vessels. Its early management before, during, and immediately after arterial reperfusion has a repercussion in the final fate of the ischemic tissue and the clinical outcome.

## Introduction

Blood pressure is usually elevated in the acute phase of all types of hemorrhagic and ischemic strokes. This acute hypertensive (presumably physiologic) response is common in the early phase of acute ischemic stroke. Approximately two-third of the ischemic strokes present with elevated systolic and diastolic blood pressure [1]. The significance of this type of hemodynamic response is unclear. In large vessel occlusion, the acute hypertensive response is believed to be responsible for the maintenance of the retrograde collateral leptomeningeal circulation. The optimal management of the blood pressure in this scenario includes balancing the risk of inappropriate lowering of the blood pressure that can jeopardize the ischemic penumbra and the excessive arterial hypertension that can promote hemorrhagic transformation of the ischemic tissue. Unfortunately, the best strategy for the management of blood pressure in patient with acute ischemic stroke is not yet established. In this review article we will focus on the latest evidence pertaining blood pressure reduction in the early phase of an acute ischemic stroke and the management of the pre- and post-operative hemodynamic response in patient with acute ischemic stroke due to large vessel occlusion undergoing to mechanical thrombectomy.

## Natural History of the Acute Hypertensive Response in Acute Ischemic Stroke

After an acute occlusion of a major intracranial artery, the cerebral tissue is able to sense a decrease in the interstitial oxygen tension [2]. The resulting ischemia around the cerebral tissue is able to trigger a glio-neurovascular signal that produces an increased sympathetic outflow from the central nervous system into the cardiovascular system that induces an elevation of the vascular tone [3]. This explains in part why subjects without premorbid arterial hypertension can experience severe elevation of the systolic and diastolic blood pressure minutes after suffering an embolic occlusion of a proximal intracranial artery. The magnitude of the hypertensive response tends to correlate with the size of tissue at risk. Large areas of ischemic penumbral cerebral tissue can provoke more dramatic acute hypertensive response. This neuroendocrine response can also be augmented by undiagnosed or untreated premorbid systemic arterial hypertension [4].

The acute hypertensive response observed in the acute phase of ischemic stroke is self-limiting, and it tends to decline over the course of the next several days and return to the premorbid baseline levels. In embolic strokes, this tends to happen at the same time when spontaneous recanalization occurs. Mechanical thrombectomy for large vessel occlusion can shorten this period, and it is not uncommon to observe a dramatic reduction of the systolic blood pressure after the vessel is recanalized. On the contrary, in cases of ineffective vessel recanalization, the acute hypertensive response may persist for several days.

## Impact of Early Lowering of the Blood Pressure Across All Acute Ischemic Strokes

Sudden and aggressive reduction of the blood pressure is deleterious across all types of ischemic strokes. This is also true in intracerebral hemorrhage. In the case of hypertensive intracerebral hemorrhage, two different randomized clinical trials showed a tendency for reduction in the hematoma expansion with early and moderate reduction of the hypertensive response [5, 6]. However, more aggressive reduction in the blood pressure not only did not result in further reduction of the hematoma expansion but also resulted in adverse complication related to cerebral, , and cardiac hypoperfusion. Unfortunately, the timing, intensity, and the duration of the correction of the acute hypertensive response in both ischemic and hemorrhagic forms of stroke is not yet determined.

Less than one-third of acute ischemic stroke can present without elevation of the blood pressure, and some of the can present with low blood pressure level. Subjects presenting with acute ischemic stroke and lack of acute hypertensive response can harbor other cardiovascular comorbidities including concomitant congestive heart failure and severe aortic and mitral valvular disease. These patients are well known to face worse outcomes in spite of the successful acute interventions. In a recent secondary analysis of the Head Positioning in acute Stroke Trial (HeadPoST), patients with acute ischemic stroke presenting initially with low blood pressure, defined as a systolic blood pressure less than 120 mmHg and a diastolic blood pressure less than 70 mmHg, had an increased risk of death or dependency (adjusted OR 1.27, 95% CI 1.02–1.58) compared with patients presenting with acute hypertensive physiologic response [7]. The association with poor clinical outcome in patients with acute ischemic stroke and failure to activate a hypertensive response persisted after adjusting for several confounding factors including baseline heart failure and cardiac disease. Importantly, these patients were at increased risk for severe adverse cardiac effects as well as worse functional outcome from their stroke.

Acute ischemic stroke patients presenting with an acute physiologic hypertensive response represent the majority of the cases. For the subset of patients arriving with the first 3–4½ h after the symptoms onset guidelines recommend to maintain the systolic blood pressure below 185 mmHg and the diastolic blood pressure below 105 mmHg. The original studies that tested the effectiveness of intravenous r-tPA for acute ischemic stroke used these blood pressure thresholds to reduce the risk of symptomatic hemorrhagic transformation that could potently offset the benefit of the thrombolytic treatment. However, this trials did not assess a specific blood pressure target for the lower limit of the goal. A U-shaped association between the initial blood pressure and the final unfavorable outcome in acute ischemic stroke was demonstrated in several observational studies. The extremes (low blood pressure and excessively elevated blood pressure) range are associated with worse outcome, and the best outcomes appear to be present with a modest initial hypertension. However, none of these observational studies were able to prove causality. Two large registries of intravenous thrombolysis in acute ischemic stroke reported the association between hypertension and the risk of the symptomatic hemorrhagic transformation [8, 9]. In both registries, the subjects presenting with systolic blood pressure higher than 170 mmHg have four more chances of symptomatic intracerebral hemorrhage as compared with subjects presenting with systolic blood pressure between 140 and 150 mmHg. In a meta-analysis of the effect of blood pressure lowering in early ischemic stroke, a total of 12,703 individuals were included, with 6392 participants randomly assigned to the active treatment group (lowering of blood pressure) and 6311 to the control group [10]. The active group underwent to modest reduction of systolic and diastolic blood pressure in the first 24 h. However, the active group did not showed any significant reduction in the risk of death or dependency at 3 months. The international, randomized, open-label, blinded-endpoint, phase 3 trial specifically assessed the potential benefit of lowering the blood pressure to reduce the risk hemorrhagic transformation during administration of intravenous Alteplase (ENCHANTED trial) [11••]. The study randomized subjects with acute ischemic stroke presenting with a systolic blood pressure equal or higher than 150 mmHg if they fulfilled the standard criteria for intravenous r-r-tPA into two groups: intensive systolic blood pressure control (130–140 mmHg) versus guideline-directed systolic blood pressure control (< 185 mmHg). The primary outcome was the percentage of death or disability at 3 months and unfortunately it did not differ between the groups. Several lessons can be learned from the ENCHANTED trial. Majority of the ischemic stroke were mild to moderate (average NIHSS score of 7 points) and even more important the difference in the systolic blood pressure between the two groups was only 7 mmHg (146 versus 153 mmHg mean systolic arterial blood pressure in the active versus the control arm). This was perhaps the main reason why the trial was negative. However, there was a clear tendency toward a lower incidence of hemorrhagic transformation particularly major intracerebral hemorrhage in the intensive blood pressure arm but unfortunately it did not improve the final primary clinical outcome. Until further evidence are available, the management of the initial arterial blood pressure in the acute setting of acute ischemic stroke triage particularly in the first three to four and a half hours after symptoms onset in subjects considered candidates for intravenous thrombolysis should follow the latest guidelines recommendations of a systolic blood pressure equal or less than 185 mmHg and a diastolic blood pressure equal or less than 105 mmHg. In the future, randomize clinical trials assessing the impact of arterial blood pressure manipulation to improve the outcome should focus in the subset of ischemic stroke in which the elevation of the systemic blood pressure may play a more prominent role in the maintenance of the collateral blood flow like in subjects with proximal large vessel occlusion amenable to endovascular recanalization.

## Management of the Blood Pressure in Acute Ischemic Stroke Before and During Endovascular Recanalization

Large vessel occlusion is perhaps the subtype of acute ischemic stroke in which the relevance of the acute hypertensive response and its adequate management are of great significance in the final outcome. The acute hypertensive response is noticeable immediately after the embolic occlusion of a proximal intracranial artery. This instantaneous systemic hemodynamic physiologic response is key for the maintenance of the retrograde leptomeningeal collateral circulation. It is also responsible of the initial minimal neurological deficit present in patient with acute large vessel occlusions presenting and low NIHSS score (equal or lower than 5 points) or even with a complete resolution of the clinical symptoms (transient ischemic attack). However, this could be a precarious situation, and it is calculated that approximately 20 to 40% of this subjects will deteriorate their neurologic condition during the subsequent 24–72 h. In this context, arterial hypotension (spontaneous or induced) well known to be obviously deleterious for the final fate of the penumbral tissue and finally the clinical outcome. Spontaneous hypotension can be seen in subjects with large vessel occlusion and concomitant cardiac disease that impairs the stroke volume. Iatrogenic systemic hypotension can be seen in patients with large vessel occlusion that undergo to mechanical thrombectomy under general anesthesia. During the induction of general anesthesia, the inhaled volatile anesthetics can cause inappropriate lowering of the systolic and mean arterial blood pressure that can overcorrect the acute hypertensive physiologic response. In a retrospective study of 371 patients that underwent to mechanical thrombectomy under general anesthesia, a linear association between arterial hypotension and worse outcome was demonstrated [12]. Even a single (less than 10%) drop from baseline resulted in worse neurologic outcome. Even single mean arterial blood pressure drop during mechanical thrombectomy under general anesthesia could result in poor neurologic outcome. In retrospective cohort study of 115 subjects that underwent to mechanical thrombectomy under general anesthesia, it was found that subjects that suffered from drops in the mean arterial blood pressure below 60 mmHg faced a worse outcome [13]. If mechanical thrombectomy will be performed under general anesthesia, the anesthesia team has to be aware that even short episodes of the hypotension during the general anesthesia induction can result in impairment of the collateral circulation. A dedicated cerebrovascular anesthesia team that can provide prompt intubation and general anesthesia in a fast and effective manner without delaying the recanalization times and without causing inappropriate lowering of the systemic blood pressure should be a critical component of comprehensive stroke centers [14]. As a general rule, patients with large vessel occlusion undergoing to mechanical thrombectomy and concomitant intravenous thrombolysis should follow the same recommendation the systolic and diastolic blood pressure parameters for intravenous rt-PA candidates (systolic blood pressure equal or less than 185 mmHg and diastolic blood pressure equal or less than 105 mmHg). In patients with large vessel occlusion undergoing to mechanical thrombectomy without concomitant intravenous thrombolysis, the systolic and diastolic blood pressure parameters should follow the blood pressure recommendation of acute ischemic stroke patients not candidate to intravenous thrombolysis (systolic blood pressure equal or less than 220 mmHg and diastolic blood pressure equal or less than 120 mmHg).

## Management of the Blood Pressure in Acute Ischemic Stroke Immediately After Successful Endovascular Recanalization

**Fig. 1:**
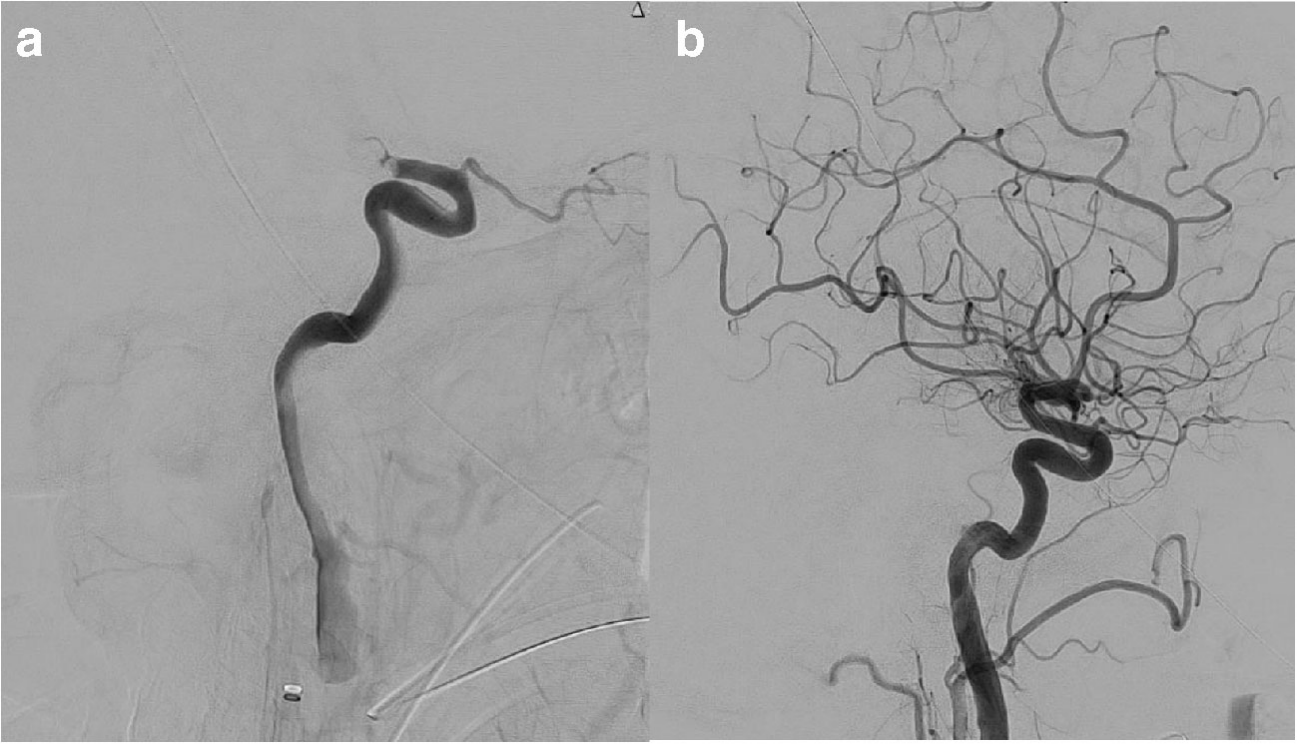
Panel **a** Lateral view from digital subtraction angiography of the brain showing an acute embolic occlusion of the right internal carotid artery terminus corresponding to a thrombolysis in cerebral ischemia score of zero. Panel **b** Lateral view from digital subtraction angiography of the brain showing complete recanalization after mechanical thrombectomy corresponding to a thrombolysis in cerebral ischemia score of three

Successful endovascular recanalization in large vessel occlusion is the most important predictor of subsequent successful clinical outcome at 90 days. A successful recanalization implies an almost complete or complete reperfusion of the occluded vessel, defined as TICI (Thrombolysis In Cerebral Ischemia) 2b or higher (Fig. 1) score. The endovascular recanalization has to happen in a timely fashion before a large infarcted tissue is established. The survival of the penumbral tissue (cerebral tissue in the process to die but not death yet) largely depend in the effectiveness of the collateral system that as we pointed before it is highly dependent of changes in the systemic blood pressure.

A successful recanalization is commonly associated with spontaneous regression and disappearance of the acute hypertensive physiologic response. The lack of spontaneous resolution of the acute hypertensive response after a successful recanalization has been correlated with worse neurologic outcome in several observational studies. The reason behind the persistence of arterial hypertension in spite of successful recanalization is probably multifactorial. Uncontrolled premorbid hypertension might explain some cases but also the fast progression to ischemia in spite of early and effective vessel recanalization is also suspected. In this last circumstance, the ischemic gliovascular tissue might continue sending a signal that keeps the sympathetic outflow from the central nervous system into the cardiovascular system. Post-operative arterial hypertension in patients that underwent to a successful recanalization has been correlated with higher chances of hemorrhagic transformation.

The impact of blood pressure levels within the first 24 h after mechanical thrombectomy on the clinical outcome was reported in a recent retrospective study that included 700 patients with large vessel occlusion that underwent to mechanical thrombectomy [15]. The study found that subjects with lower levels of blood pressure (less than 140 mmHg) 24 h after the mechanical thrombectomy tend to have better outcome and lower mortality at 3 months. It is unknown if the early correction of the persistent hypertensive response would result in better outcomes including less incidence of hemorrhagic complications. In a multicenter retrospective study of the relationship between systemic blood pressure reduction and outcome after successful reperfusion included a total of 1454 patients were included [16]. More than half of the sample underwent to a complete angiographic reperfusion (TICI: 3 score) with a mean time of onset to groin puncture of 216 min. The systolic blood pressure reduction was associated with lower odds of poor outcome. This was particularly consistent in subjects with complete recanalization and no history of premorbid hypertension. Another retrospective study from Taiwan showed that the uncontrolled elevation of the blood pressure in the immediate first 6 h after a successful mechanical thrombectomy is an important factor in the final clinical outcome [17••]. The study found that 50% of the subjects that underwent to a successful recanalization were able to be functionally independent at 90 days. In this particular group, the blood pressure levels were analyzed in the first 24 h after mechanical thrombectomy. The groups were divided into four intervals (0–6, 7–12, 22–19, and 19–24 h). There was a linear association between post-operative hypertension and worse outcome at 90 days. The association was stronger if the average highest blood pressure recording was registered in the first 6 h.

Areas of hyperatenuation in the brain parenchyma (on computerized tomography of the head obtained without intravenous injection of iodinated contrast material) represent regions of the brain tissue with broken brain blood barrier due to established ischemia after mechanical thrombectomy. This area of hyperatenuation is more prone to hemorrhagic transformation after effective recanalization. In a retrospective study of a prospectively collected cohort of the consecutive acute ischemic stroke patients due to large vessel occlusion that underwent to a successful mechanical thrombectomy, 50% of the subjects exhibited areas of the hyperatenuation in the post-procedure non contrast CT [18]. In this particular group, the probability of symptomatic hemorrhagic transformation (parenchymal hematoma) due to reperfusion injury increased with each increment of the post-operative maximum systolic blood pressure in the first 24 h.

**Fig. 2:**
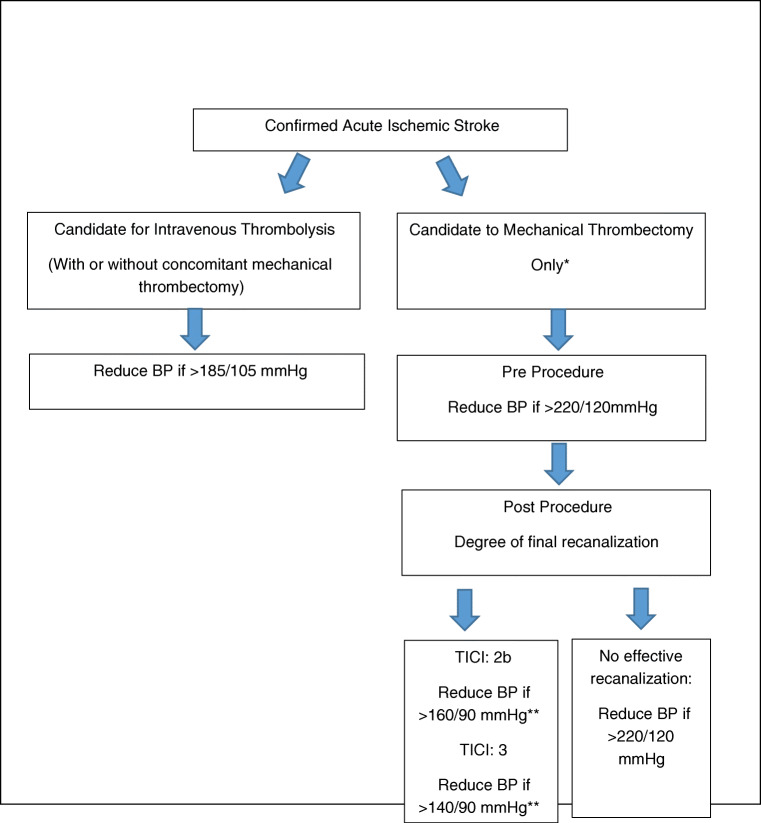
Algorithm for intervention in the acute hypertensive response in patients presenting with acute ischemic stroke considered for acute reperfusion therapies. Cautious and stepwise blood pressure reduction (no more than 20% if the baseline MAP) is advised. Placement of arterial line for continuous real time blood pressure monitoring is suggested. Intravenous bolus and intravenous infusion of short-acting vasodilators is recommended. *If mechanical thrombectomy is performed concomitant to IV rt-PA infusion then the blood pressure parameters for intravenous thrombolysis should be followed. **Consider history of premorbid arterial hypertension and baseline systolic and diastolic blood pressure

Until further evidence from randomized clinical trials are available, it appears prudent to try to correct the persistence of an acute hypertensive response after a successful mechanical thrombectomy to avoid hemorrhagic reperfusion injury. This is particularly important in subjects with early signs of established ischemia as evidenced by areas of hyperatenuation in the immediate post-operative non contrast CT of the head. Based on the best current evidence, the systolic blood pressure should range between 140 and 160 mmHg and diastolic blood pressure below 90 mmHg after successful thrombectomy. Caution should be applied with subjects with history of premorbid of arterial hypertension. The control of the blood pressure should start in the Cath lab immediately after the clot is removed. We suggest the placement of an invasive arterial line to facilitate a continuous and accurate blood pressure monitoring for at least 24–72 h as well as to facilitate the accurate titration of the doses of the intravenous short-acting vasodilators. If the Cath lab has the capability to perform flat panel detector-computerized tomography of the head, we do suggest to acquire the information of hypertanuated areas of the brain parenchyma to better identify subjects with higher risk of the reperfusion injury and implement therapeutic measurements (including blood pressure control) to improve the final clinical and neurologic outcome (Fig. 2).

## Conclusion

Acute hypertensive response is considered a physiologic hemodynamic reaction of the cardiovascular system that is commonly seen in patient with acute ischemic stroke. The lack of presence of this physiologic hemodynamic response or its iatrogenic over correction has a negative impact in the final neurologic outcome, particularly in subject with large vessel occlusion before recanalization. The persistence of the hypertensive response after effective recanalization appears to negatively affect the final outcome, and the cautious lowering of the blood pressure to a safer range appears to be reasonable.
